# Mechanical properties of an elastically deformable cervical spine implant

**DOI:** 10.1186/s13018-023-04042-7

**Published:** 2023-08-16

**Authors:** Haimiti Abudouaini, Tingkui Wu, Yang Meng, Beiyu Wang, Hao Liu

**Affiliations:** 1 Department of Orthopedic Surgery, West China Hospital, Sichuan University, No. 37 Guo Xue Xiang Rd., Chengdu, China; 2 Department of Spine Surgery, Honghui Hospital, Xi’an Jiaotong University, Xi’an, China

**Keywords:** Mechanical, Cervical implant, Polyurethane, Elastic deformation

## Abstract

Anterior cervical surgery is widely accepted and time-tested surgical procedure for treating cervical radiculopathy and myelopathy. However, there is concern about the high adjacent segment degeneration rate and implant subsidence after the surgery using the traditional polyetheretherketone cage. Thus, we creatively designed a polyurethane cervical implant that can continuous load-sharing through elastic deformation and decrease postoperative stress concentration at adjacent segments. In this study, the design rationality and safety of this novel implant was evaluated based on several mechanical parameters including compression test, creeping test, push-out test and subsidence test. The results showed that the novel cervical implant remained intact under the compressive axial load of 8000 N and continues to maintained the elastic deformation phase. The minimum push-out load of the implant was 181.17 N, which was significantly higher than the maximum compressive shear load of 20 N experienced by a normal human cervical intervertebral disc. Besides, the creep recovery behaviour of the implant closely resembled what has been reported for natural intervertebral discs and clinically applied cervical devices in literature. Under the load of simulating daily activities of the cervical spine, the implant longitudinal displacement was only 0.54 mm. In conclusion, this study showed that the current design of the elastically deformable implant was reasonable and stable to fulfil the mechanical requirements of a cervical prosthesis under physiological loads. After a more comprehensive understanding of bone formation and stress distribution after implantation, this cervical implant is promising to be applied to certain patients in clinical practice.

## Introduction

Anterior cervical discectomy and fusion (ACDF) is considered to be the most popular and well-studied treatment for patients with cervical disc disease who have failed conservative treatments [1]. However, by converting a mobile, functional spinal unit into a fused segment, ACDF has been shown to increase motion and strain at adjacent levels, leading to accelerated disc degeneration [2, 3]. As a suitable alternative to ACDF, anterior cervical disc replacement (ACDR) effectively maintains the segmental range of motion (ROM) and reduces the risk of adjacent level disc degeneration. Finite element studies and biomechanical analyses have confirmed that cervical arthroplasty devices preserve normal motion, disc stresses, and facet loading at the adjacent levels [4–6]. However, the surgical indications for ACDR are particularly narrow. At present, although there are no unified criteria for the indications of ACDR, these criteria can be summarized as mild-to-moderate cervical intervertebral disc disease with certain degrees of preoperative segmental motion [4, 7–9].

When the human cervical spine is under the application of forces, the cervical intervertebral disc will undergo elastic deformation, distribute the load to the surrounding areas, absorb the concussion of the spine exerted by the external forces, and serve a stress-buffering role. Currently applied titanium and polyetheretherketone (PEEK) cervical intervertebral cages have not well-mimicked the resilience and cushioning performance of normal cervical discs, and it is difficult to effectively distribute the load, which can easily lead to stress concentrations. Elastic polymeric materials exhibit special absorbed compressional energy performance when subjected to mechanical impact and compression, which provides them with certain advantages in mimicking the cushioning and shock absorption functions of the normal intervertebral disc. Consequently, polymeric implants can act as shock absorbers and possibly reduce stress at adjacent segments.

Thus, we creatively designed a novel cervical implant with enhanced cushioning and shock absorption functions combined with personalized morphology. The design concept of this new cervical implant allows for the provision of satisfactory stabilization and appropriate deformation can occur to dynamically adapt to the stress variation which can thereby reduce adjacent segment stress and maintain the operated segment intervertebral height. Therefore, the purpose of this study was to evaluate the mechanical parameters through compression test, creeping test, push-out test and subsidence test. The results of this study may provide a theoretical basis for further improvements of this novel cervical implant, as well as further animal studies and human clinical applications.

## Materials and methods

### Implant design

**Fig. 1:**
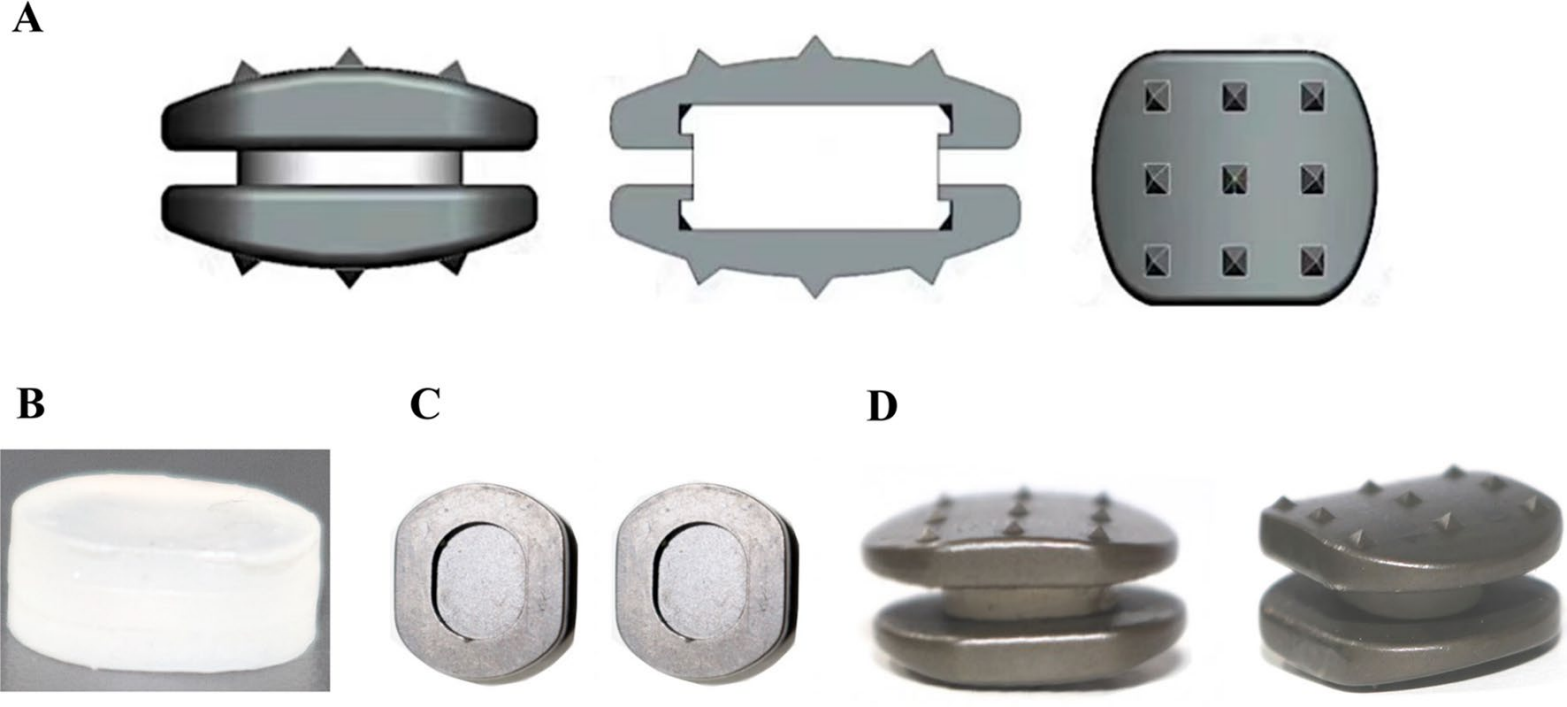
The manufacturing process of the novel cervical implant: **A** a 3D plot of the implant was generated using Pro-Engineer software prior to manufacturing, and **B** cylindrical elastomer with 35% hard segment polyurethane was synthesized. Then, **C** upper and lower titanium alloy plate was prepared using the selective laser sintering (SLS). **D** The elastomer and titanium alloy plate were fixed by using a mortise and tenon connection, and preparation of the implant was completed

The novel cervical implant consists of an elastomeric structure with cushioning properties and a titanium alloy (Ti-6Al-4V), and they were fixed by using a mortise and tenon connection (Fig. 1). Among them, the elastomer is fabricated from 35% hard segment polyurethane. The implant was designed to allow continuous load-sharing through the entire range of motion of the cervical spine. The 1,6- polycarbonate diol (PCDL, Ube Industries Ltd., Ube, Japan) was used as a soft segment, and 4,4′-methylene diphenyl diisocyanate (MDI, Wanhua Company, Yantai, China) and 1,4-butanediol (BDO, Kelong Chemical Reagent Company, Chengdu, China) chain extenders were used as hard segments (Fig. 1). The elastomer was synthesized in two steps. First, the PCDL was heated to 105 °C, dehydrated in a vacuum oven for 2 h, and then cooled to 45–50 °C under vacuum conditions, after which appropriate amounts of melting were added to fully transparent MDI fluid for prepolymerization. These prepolymer solutions were reacted at 78–80 °C for 100–120 min. After prepolymerization, prepolymer solutions were cooled to 40–45 °C, and appropriate amounts of BDO were added and chain-extended for 2–3 min under vacuum to strengthen its flowability. Afterwards, they were poured into a prepared mould and finally matured in a 120 °C oven for 4–6 h (Fig. 1B). In addition, the selective laser sintering (SLS) was used to prepare the titanium alloy plate of the prosthesis. For preprocessing, the 3D CAD model exported from Geomagic Studio software (3D Systems, USA) was converted into STL data via the Mimics Medical software (version 19.0, Leuven, Belgium) and was input into a laser sintering system. The surface of the titanium alloy plate was subjected to microarc oxidation to further improve its strength and wearability (Fig. 1C).

### Compression test

**Fig. 2:**
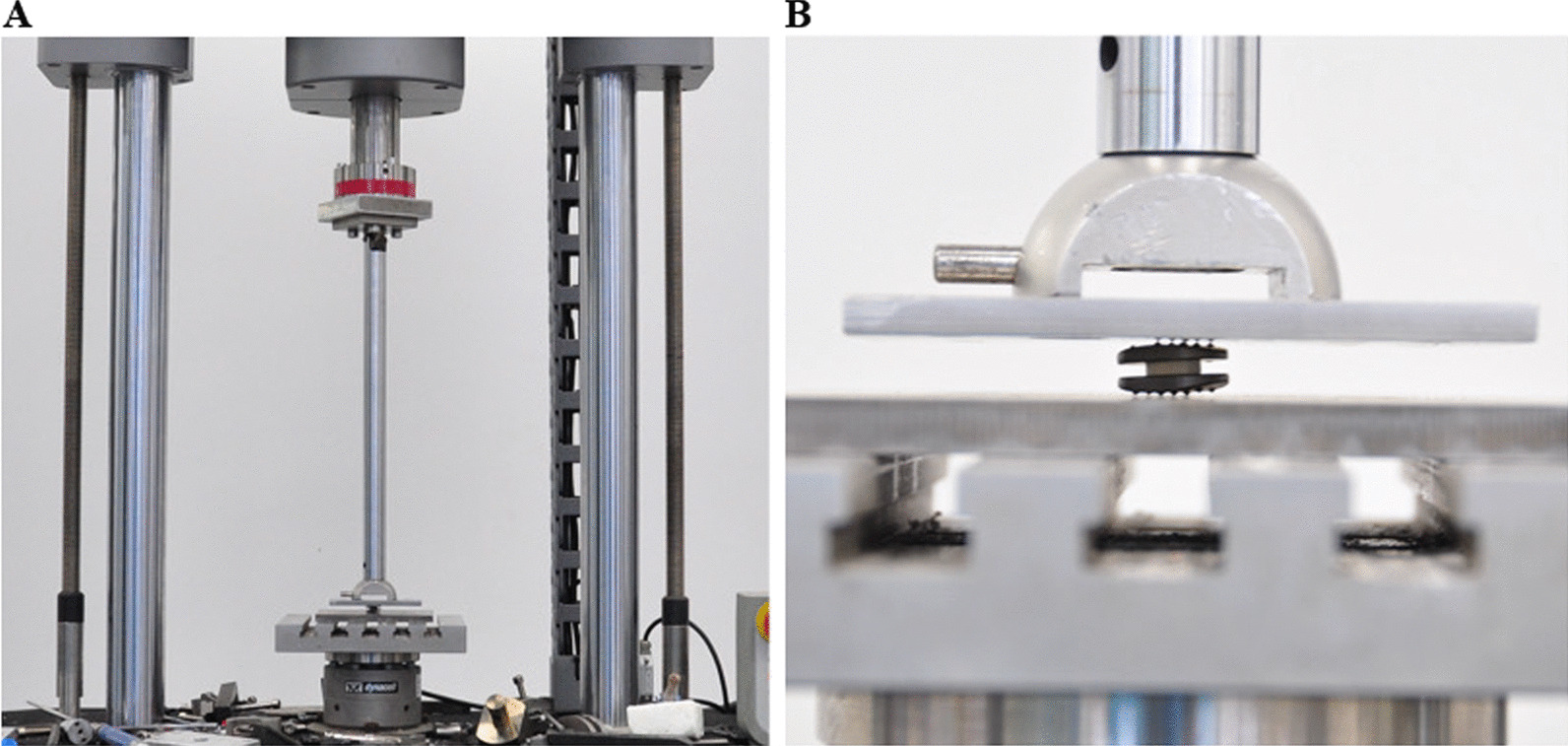
The compression test was performed using the electronic universal testing machine (CMT7504, SANS, USA) according to the ASTM F2346-05

The compression test was conducted according to the ASTM F2346-05, and the testing loadings ranged from 0 to 8000 N (Fig. 2). The novel cervical implant was measured under a strain rate of 5 mm/min three times, and the average of the results was taken. The laser displacement sensor continuously recorded the changes in displacement and loading. Subsequently, the result was analysed, and a load‒displacement curve was drawn.

### Creeping test

**Fig. 3:**
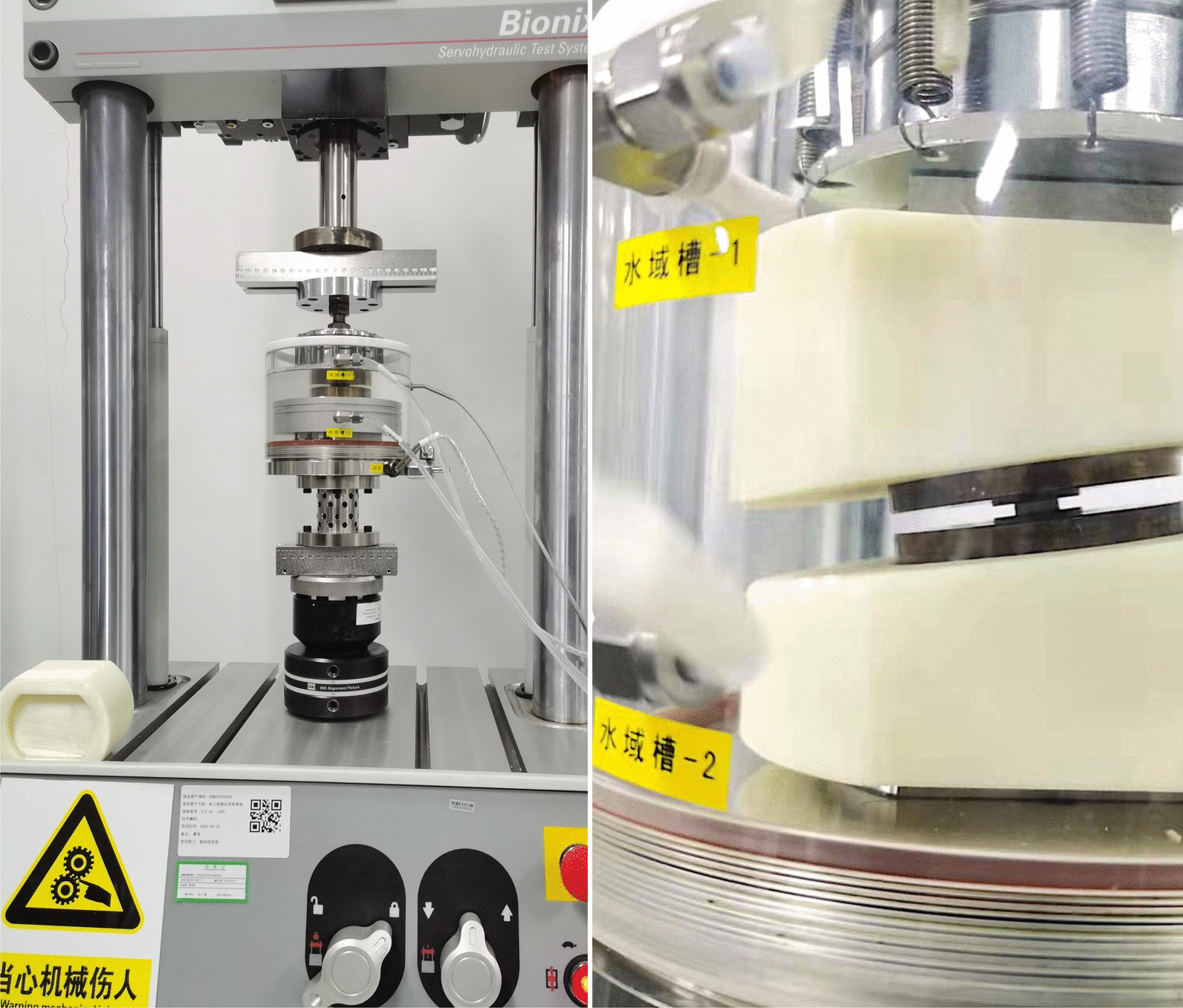
The creeping test was performed on a biaxial servo-hydraulic testing machine MTS Mini Bionix II 858 (MTS Systems Corp., USA) with 3600 N load for 24 h

First, the novel cervical implant was fixed onto the adapter of the material testing system and immersed in simulated body fluid (SBF) at 37 °C (Fig. 3). Afterwards, a continuous small static and large dynamic load was applied. The maximum displacement (mm) was recorded, and the corresponding displacement under the physiological loading was calculated.

### Push-out test

**Fig. 4:**
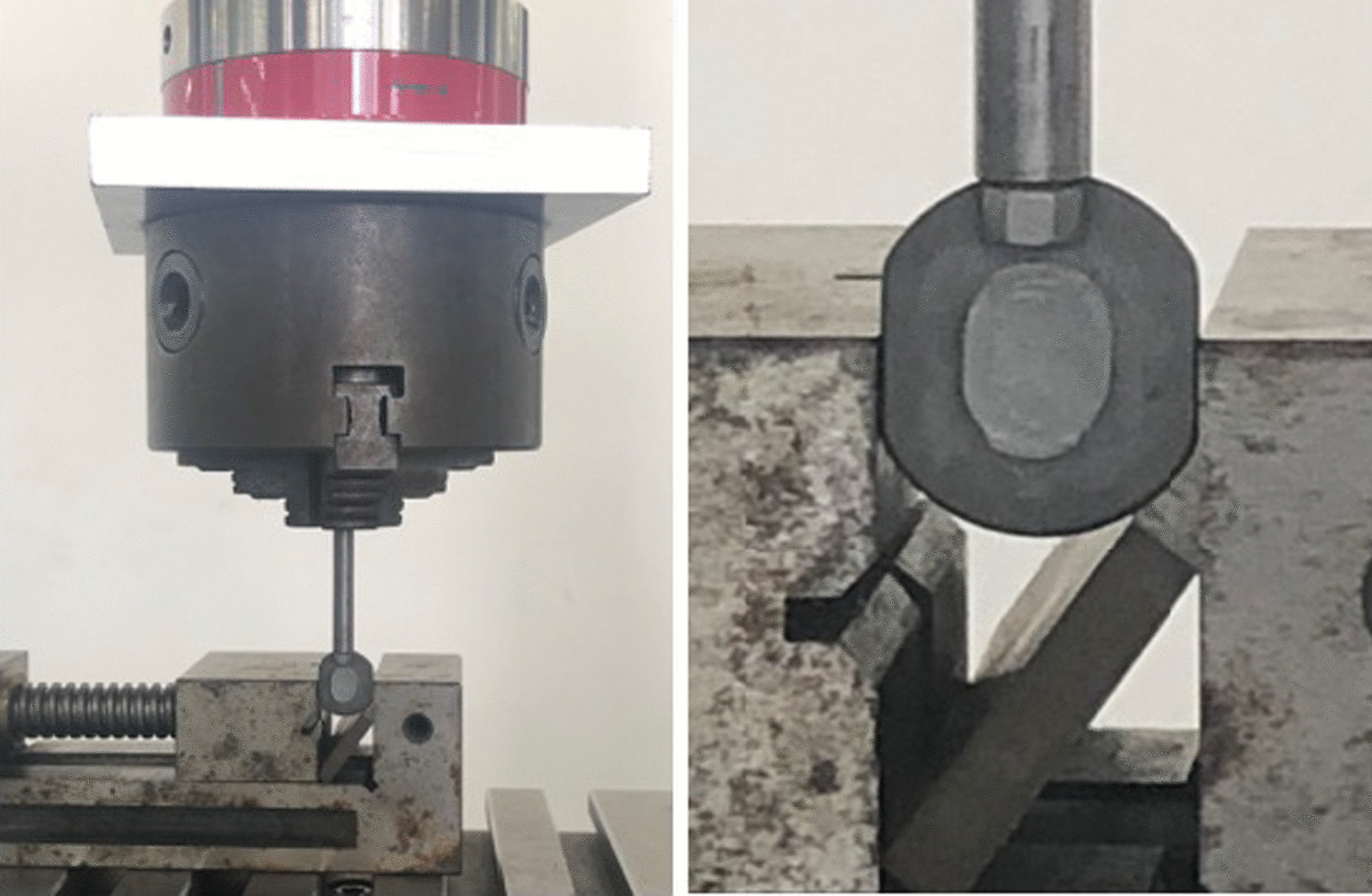
The implant was fixed in the Instron Universal Testing machine (E3366, Instron, MA, America), and additional gradually increased lateral shear load was applied until the elastomer structure exceeded the setting displacement

The elastomer structure was installed in a lower titanium alloy plate, and a continuous unchanged axial load. According to industry standards ASTM F2346, the loading rate should not exceed 25 mm/min. Therefore, the 10 mm/min was selected as a consistent speed (Fig. 4). Afterwards, an additional gradually increased lateral shear load was applied until the elastomer structure exceeded the setting displacement.

### Subsidence test

**Fig. 5:**
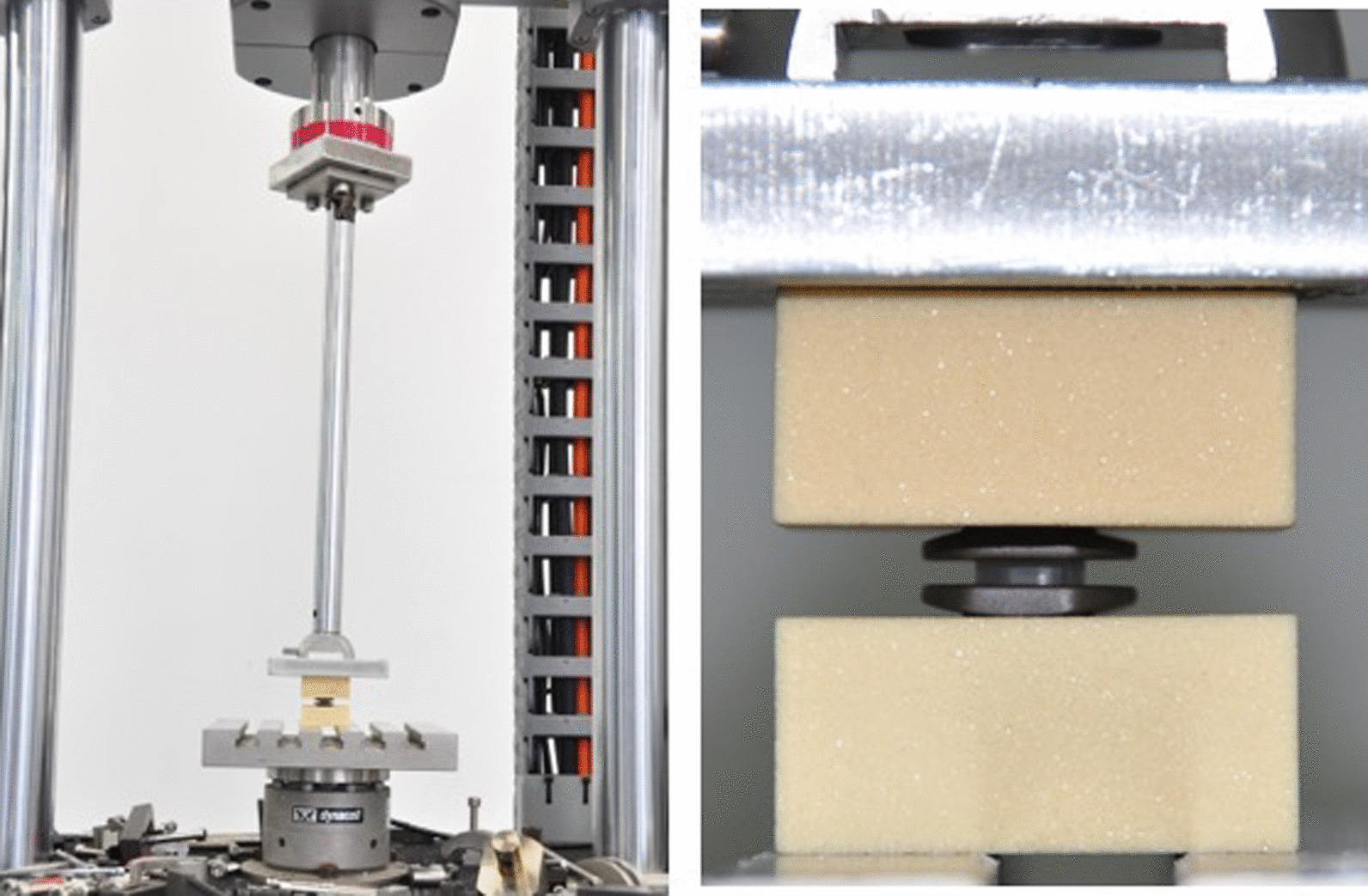
The subsidence test was performed with a microcomputer control electronic universal testing machine (CMT7504; SANS, USA). The implant was placed on the simulated bone, and an gradually increased axial load was applied

For the simulation of the postimplantation intervertebral state, the novel cervical implant was placed on the simulated bone, and a gradually increased axial load was applied (Table 1, Fig. 5). The subsidence displacement was recorded and compared with other cervical implants [10–14]. If the axial displacement was large, it indicated that the potential risk of implant subsidence occurrence was high.

**Table 1:** The detail characteristics of simulated bone used in the subsidence test

Rank (PCF)	15
Density (Kg/m^3^)	240.3
Compressive strength (Mpa)	3.820–6.050
Compressive modulus (Mpa)	98.00–151.0
Shear strength (Mpa)	2.235–3.510
Shear modulus (Mpa)	27.10–39.70

### Statistical analysis

We performed statistical analysis with SPSS software (version 25.0, IBM Corp). Categorical variables are summarized as percentages, and continuous variables are summarized as the mean ± standard deviation (SD). A two-sided *p* value of < 0.05 was considered statistically significant.

## Results

### Compression test

**Fig. 6:**
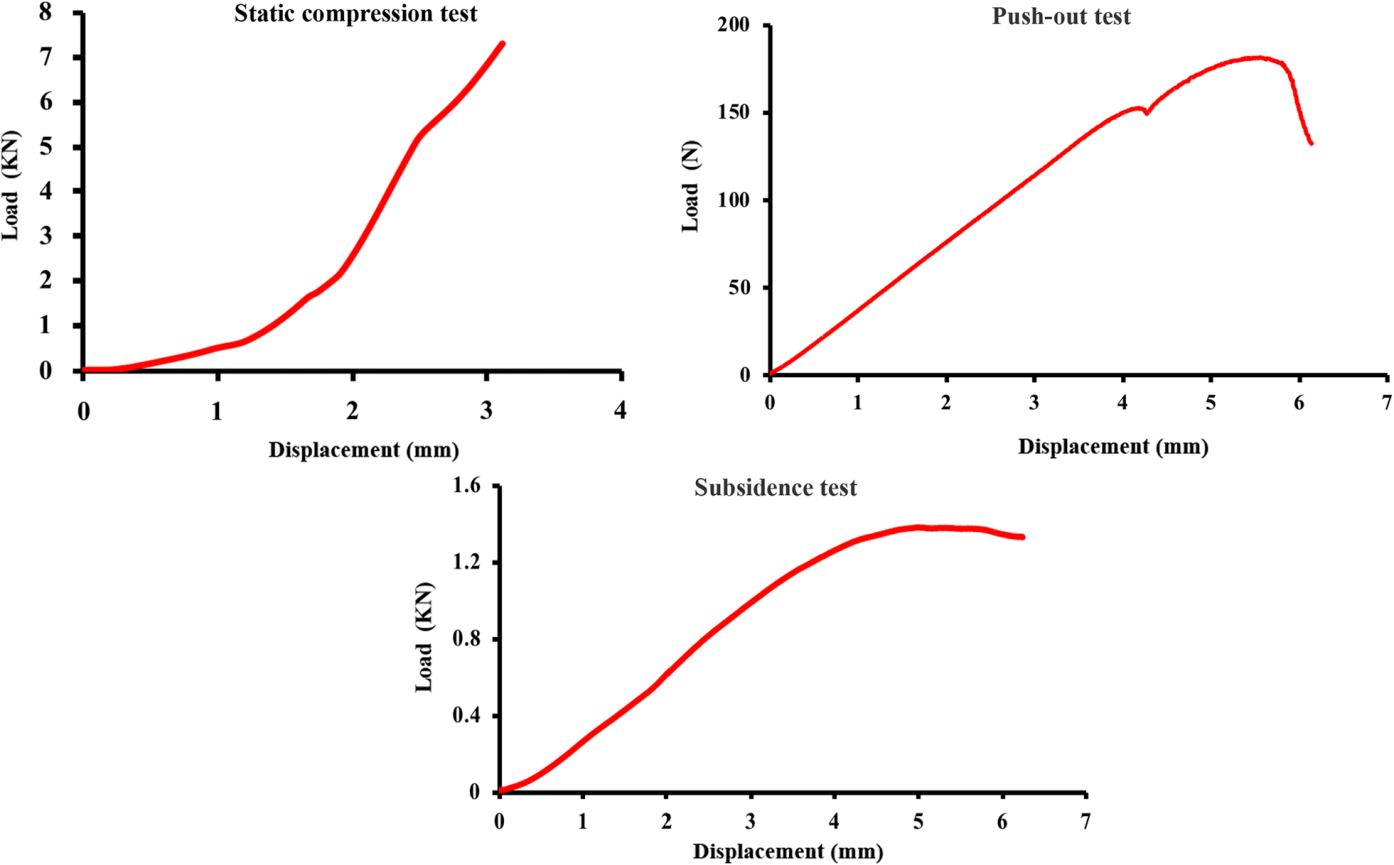
The results of the static compression test, push-out test and subsidence test. The results indicated that the structural design of the novel cervical implant was stable and fulfilled the mechanical property requirements of the cervical prosthesis

The load‒displacement curve of the static compression test is shown in Fig. 6A. During the entire test procedure (0–8000 N), the novel cervical implant was in the elastic deformation stage, and the failure of the prosthesis was not observed.

### Creeping test

As shown in Table 2, the result of the creeping test confirmed that after 24 h of axial compression with 3600 N, the novel cervical implant had a permanent deformation amount lower than 0.63 mm. The creep recovery behaviour of the implant closely resembled what has been reported for natural intervertebral discs and clinically applied cervical devices in literature [10–14].

**Table 2:** Comparison of creeping test results of each experimental group (mm)

Sample serial number	Initial height	Height after creep deformation	Height change
First	8.301	7.569	0.732
Second	8.262	7.733	0.529
Third	8.418	7.783	0.635
Average value	8.327	7.695	0.632

### Push-out test

The results of the push-out test confirmed that the minimal push-out force was 181.17 N, which was far greater than the maximum shear force (20 N) suffered by normal human cervical intervertebral discs [15, 16]. This result indicated that the novel cervical implant had good stability (Fig. 6B).

### Subsidence test

The results of the subsidence test confirmed that the yield load of the novel cervical implant was 1.38 KN, the corresponding longitudinal displacement was 4.988 mm, the maximum load on the cervical spine of the human body was 150 N [17], and the implant longitudinal displacement was 0.54 mm. This result indicated that the risk of subsidence of the novel cervical implant was relatively low (Fig. 6C).

## Discussion

The mechanical properties of the cervical implant are important parameters for practical applications. Through in vitro functional testing, the design rationality and safety of prosthesis designs can be effectively evaluated. The results of the compression test showed that during the entire test procedure (0–8000 N), the novel cervical implant was in the elastic deformation stage, and the failure of the prosthesis was not observed. The Food and Drug Administration (FDA) has published compression test results for 4 types of artificial cervical disc prostheses [11–14]. Among them, the yield loads of the Mobi-C, SECURE-C, and Prestige LP cervical disc prostheses were 1,934.98 N, 1,677 N, and 7992 N, respectively [11–14]. Therefore, the compressive properties of the novel cervical implant were superior to those of the Mobi-C, Prestige LP, and SECURE-C cervical disc prostheses.

According to the stress duration of cervical implants, the stress can be divided into short duration, high intensity, large-load stress and long duration, low intensity, small-load stress. For cervical implants involving polymer materials, due to the viscoelasticity of the polymers, the maintenance of intervertebral height is related to loading time. Creeping and relaxation of the polymer materials may occur over time, thus resulting in secondary intervertebral height loss and impact between the upper and lower titanium alloy plates. Therefore, we conducted a creeping test for the novel cervical implant. The result of the creeping test confirmed that the novel cervical implant had a permanent deformation amount lower than 0.63 mm. Previous studies have reported that the permanent deformation amount was 0.8 mm for the Mobi-C and 0.469 mm for the ProDisc-C cervical disc prosthesis [11–14]. Thus, the novel cervical implant fulfilled the requirements of the anti-creep deformation ability of the cervical prosthesis.

Due to the fact that the novel cervical implant is composed of upper and lower titanium alloy plates and an intermediate elastomer, there is potential risk that the elastomer eluted from the upper and lower plates. The structural strength of the novel cervical implant was evaluated by using the push-out test. In addition, the incidence of implant subsidence after cervical surgery is still high. Although previous studies have reported the related risk factors for implant subsidence, the occurrence of subsidence did not significantly decrease and still fluctuates between 15 and 30% [18–21]. The results of the subsidence test confirmed that under a loading force of 150 N, the implant longitudinal displacement was 0.54 mm. The implant longitudinal displacement was 0.44 mm for the PCM prosthesis and 0.45 mm for the Mobi-C prosthesis [11–14]. Although the longitudinal displacement of the novel cervical implant was 0.09–0.1 mm higher than that of the artificial cervical disc, the gap was not substantial. Simultaneously, the risk of implant subsidence was greatly decreased compared to intervertebral fusion devices.

The polyurethane (PU) is an important subclass of the family of thermoplastic elastomers. Due to their elastomeric behaviour, PU can find various applications in soft-tissue engineering [22–24]. Biomechanically, the elastomer used in this study is unique in that it facilitates continuous load-sharing through elastic deformation and absorb interface concussion. Polyurethane exhibit special absorbed compressional energy performance when subjected to mechanical impact and compression, providing them with certain advantages in mimicking the cushioning and shock absorption functions of the normal intervertebral disc. Gonzalez et al. [25] also reported that elastomeric lumbar disc replacement with PU could excellently mimic the axial compliance of the spine.

Several limitations of the present study should be stated. First, the chemical and physical properties of the elastomer has not been deeply explored in this study. It is difficult to report all aspects of this novel cervical implant in one study. The main purpose of this study is to observe the design rationality and safety of this cervical implant by evaluating the several mechanical parameters. Second, although all tests were conducted in accordance with industry standards or previous literature, there are still differences between experimental conditions and actual biomechanical environments. Therefore, the results of these tests may not fully represent real clinical scenarios. However, the current study aimed to provide a trend instead of natural status. Third, due to the fact that this study primarily focused on the mechanical properties of the novel implant, substantial additional work is still required to observe the bone formation and distribution of stress concentration after implantation, and if there are noticeable drawbacks in the next biomechanical experiment, the implant needs to be further improved in terms of structural design and material application.

## Conclusion

In conclusion, this study showed that the current design of the elastically deformable implant was reasonable and stable to fulfil the mechanical requirements of a cervical prosthesis under physiological loads. After a more comprehensive understanding of bone formation and stress distribution after implantation, this cervical implant promising to be applied to certain patients in clinical practice.
